# Microwave-Assisted Synthesis of Novel 2*H*-Chromene Derivatives Bearing Phenylthiazolidinones and Their Biological Activity Assessment

**DOI:** 10.3390/molecules191219648

**Published:** 2014-11-26

**Authors:** Islam H. El Azab, Mohamed M. Youssef, Mahmoud A. Amin

**Affiliations:** 1 Chemistry Department, Faculty of Science, Aswan University, Aswan 81528, Egypt; 2 Chemistry Department, Faculty of Science, Taif University, Al-Haweiah, P.O. Box 888, Taif 21974, Saudi Arabia; 3 Chemistry Department, Faculty of Science, Cairo University, Giza 12613, Egypt; 4 Chemistry Department, Faculty of Science, Suez Canal University, Ismailia 41522, Egypt

**Keywords:** multicomponent reactions (MCRs), green synthesis, microwave irradiation, 2-*H*-chromene, thiazolidenone, biological activity

## Abstract

6-Hydroxy-2-oxo-2*H*-chromene-4-carbaldehyde (**2**), 6-chloro-2-oxo-2*H*-chromene-4-carbaldehyde (**3**) and 6-hydrazinyl-4-methyl-2*H*-chromen-2-one (**5**) were prepared as single-pharmacophore motif key intermediates. Compounds **2**, **3** and **5** were incorporated in a series of multicomponent reactions (MCRs), under microwave assistance as well as conventional chemical synthesis processes, to afford a series of three and/or four-pharmacophoric-motif conjugates **8a**,**b**, **11**, **13**, **16**, **17**, **19** and **20** in good yields. The newly synthesized compounds were characterized by IR, NMR, ^13^C-NMR, MS and elemental analyses. Finally the synthesized compounds have been screened for their biological activity whereupon they exhibited remarkable antimicrobial activity on different classes of bacteria and the fungus.

## 1. Introduction

Green chemistry is a new and rapidly emerging field of chemistry. Its growing importance is in utilization of the maximum possible resources in such a way that, there is negligible or minimum production of chemical waste. It is one of the best alternatives for traditional chemical synthesis processes. By applying the green synthesis method, we can not only avoid the use of hazardous, toxic solvents, but also the formation of by-products is avoided. Thus, they are perfectly amenable to automation for combinatorial synthesis [1]. In 1986, Gedye and Giguere reported for the first time that organic reactions could be conducted very rapidly under microwave irradiation.

Coumarins are a group of compounds that play important roles as food constituents, antioxidants, stabilizers and immunomodulatory substances, as fluorescent markers for use in analyses, in stains, and in clinical use for their [2,3] diuretic [4], anti-coagulant, anti-cancer [2], anti-HIV [5], antitumor [6], anti-inflammatory [7], anti-Alzheimer’s [8], anti-leukemic [9,10], antibacterial [11], anti-malarial activities [12], emetic [13], and anti-anaphylactic activities [14]. Moreover, they can also be employed as cosmetics and pigments [13] and utilized as potential biodegradable agrochemicals [15]. Some of these compounds have been already prepared in the presence of piperidine [16], diammonium hydrogen phosphate (DAHP), *S*-proline [17], K_2_CO_3_ under microwave irradiation [18], H_6_P_2_W_18_O_62_·18H_2_O [19], MgO [20] and tetrabutylammonium bromide (TBAB) [21]. Each method has its own advantages and disadvantages.

Finally, as third motif in this preface, 4-thiazolidinones are among the most common and important groups among the small ring heterocyclic compounds. There are many references reported in the literature highlighting their chemistry and uses. 4-Thiazolidinones exhibit various biological activities such as analgesic, antibacterial, antifungal, anti-oxidant, anti-inflammatory, anticonvulsant, anticancer, anti-HIV, anti-tubercular and anthelmintic properties [22,23,24,25,26].

In connection with our previous work [27,28,29,30,31] on the synthesis of heterocyclic compounds, in the present study we describe the preparation of some new phenylthiazolidinone derivatives and heterocyclic bases from 6-hydroxy-4-methyl-2*H*-chromen-2-one.

## 2. Results and Discussion

### Chemistry

**Scheme 1:**
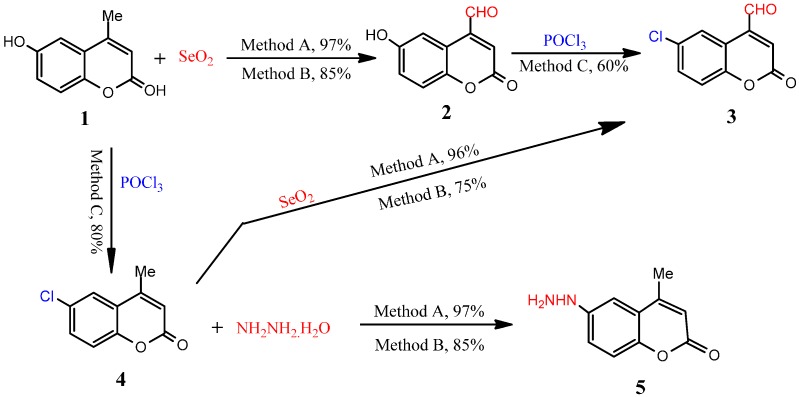
Synthesis of 2*H*-chromen-2-one derivatives **2**–**5**.

**Scheme 2:**
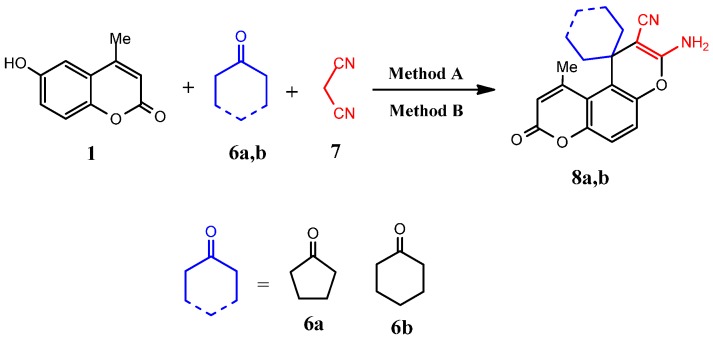
Synthesis of spiro[cycloalkane-1,1'-pyrano[3,2-*f*]chromene]-2'-carbonitriles **8a**,**b**.

**Scheme 3:**
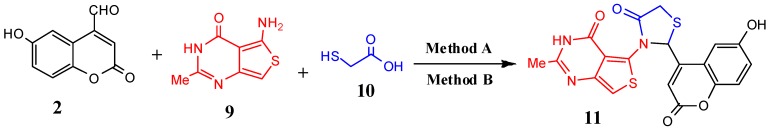
Synthesis of 5-(2-(6-hydroxy-2-oxo-2*H*-chromen-4-yl)-4-oxothiazolidin-3-yl)-2-methylthieno[3,4-*d*]pyrimidin-4(3*H*)-one (**11**).

**Scheme 4:**
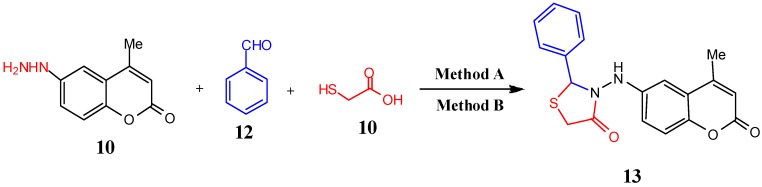
Synthesis of 3-(4-methyl-2-oxo-2*H*-chromen-6-ylamino)-2-phenylthiazolidin-4-one (**13**).

**Scheme 5:**
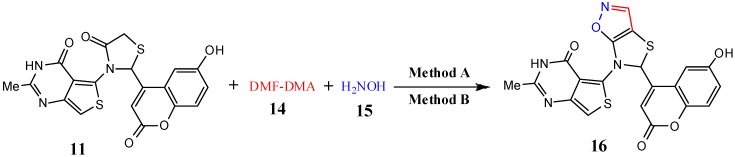
Synthesis of thiazolo[5,4-*d*]isoxazole derivative **16**.

**Scheme 6:**
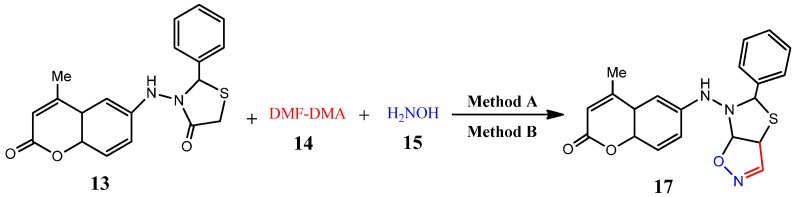
Synthesis of 4-methyl-6(5-phenylthiazolo[5,4-*d*]isoxazol-6-ylamino)-4a,8a-dihydro-2*H*-chromen-2-one (**17**).

**Scheme 7:**
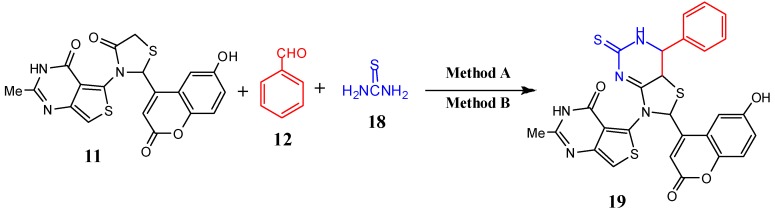
Synthesis of 7-phenyl-5-thiazolo[4,5-*d*]pyrimidine derivative **9**.

**Scheme 8:**
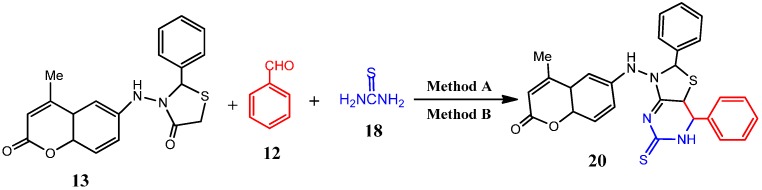
Synthesis of 2,7-diphenyl-5-thioxothiazolo[4,5-*d*]pyrimidine derivative **20**.

The synthetic strategies adopted for the synthesis of the intermediate and target compounds are depicted in Scheme 1, Scheme 2, Scheme 3, Scheme 4, Scheme 5, Scheme 6, Scheme 7 and Scheme 8. A one-pot, microwave assisted reaction condition was applied as well as conventional synthesis, by using 6-hydroxy-4-methyl-2*H*-chromen-2-one (**1**) in DMF containing 3–4 drops of glacial AcOH and selenium dioxide to give compound (**2**) (Scheme 1, Table 1). The latter compound was easily chlorinated via treatment with phosphoryl chloride in anhydrous EtOH yielding 6-chloro-2-oxo-2*H*-chromene-4-carbaldehyde (**3**) (Scheme 1, Table 1). The hydroxyl group in compound **1** was easily transformed into a chlorine to afford the 6-chlorocoumarin derivative **4**, which, in turn was reacted with hydrazine hydrate in anhydrous EtOH to give 6-hydrazinyl-4-methyl-2*H*-chromen-2-one (**5**) (Scheme 1, Table 1). The IR spectrum of **2** showed the presence of absorption bands at 1695, 1710 and 3431 cm^−1^ due to (2 C=O*_str_*) and (O–H*_str_*) functions respectively. Its ^1^H-NMR spectrum showed three singlet signals corresponding to the coumarin-C3, formyl and hydroxyl protons at δ 6.70, 10.45 and 12.01 ppm, respectively, and aromatic protons in the 7.70–7.98 ppm region, while the ^13^C-NMR spectrum of **2** showed the following signals: 91.1 (coumarin-C3), 119.8, 125.7, 126.6, 128.8, 133.7 and 150.1 (Ph), 162.4 (C=O), 192.5 (CHO). The mass spectrum of **2** displayed an intense ion peak at *m/z* 190 (M‏^+^, 51%) corresponding to C_10_H_6_O_4_. The structure of **3** was established on the basis of its elemental analyses and spectral data, as well as its independent synthesis via oxidation reaction of chlorocoumarin derivative **4** with selenium dioxide which afforded a product identical in all aspects (mp and IR spectra) with that obtained previously from the reaction of **2** with phosphoryl chloride. The IR spectra of compounds **3** and **4** do not show any absorption bands corresponding to O–H groups while they show absorption bands at 1695–1710 cm^−1^ due to C=O*_str_* functions. The mass spectrum of **4** showed a molecular ion peak at *m/z* 194 corresponding to its molecular formula (C_10_H_7_ClO_2_). The mass spectrum of **5** showed a molecular ion peak at *m/z* 190, corresponding to a molecular formula C_10_H_10_N_2_O_2_. Its ^1^H-NMR spectrum displayed new signals representing a hydrazide structure that appeared at 4.28 (–NHNH_2_) and 9.21 (–NHNH_2_) ppm (exchangeable with D_2_O) integrating for two protons and one proton, respectively.

**Method A**: Microwave-assisted synthesis: AcOH, DMF as a solvent, 120 °C, 8–10 min; **Method B**: Conventional synthesis: AcOH, DMF as a solvent, reflux 4–7 h; **Method C**: Conventional synthesis: Anhydrous EtOH as a solvent, stirring at r.t. for 1 h, then refluxes for 2 h at 60 °C.

**Chart 1:**
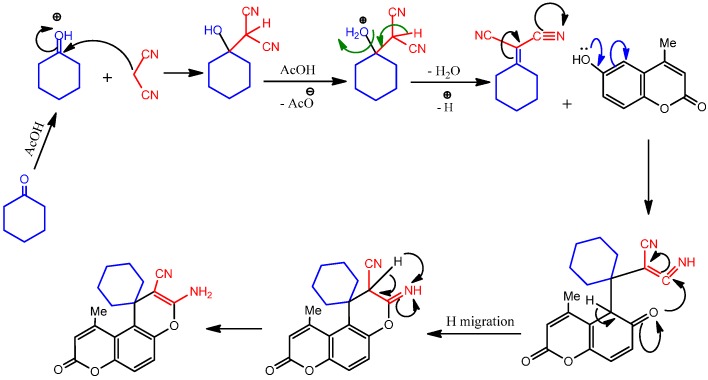
Mechanistic pathway of 3'-amino-10'-methyl-8'-oxo-8'*H*-spiro-[cyclohexane-1,1'-pyrano-[3,2-*f*]chromene]-2'-carbonitrile **8b**.

Spiro compounds represent an important class of naturally occurring molecules characterized by highly pronounced biological properties [32]. In this context, we explored the synthetic versatility of 6-hydroxy-4-methyl-2*H*-chromen-2-one (**1**) for the synthesis of spiro compounds containing the coumarin moiety. Thus, a one-pot, three-component, microwave assisted reaction condition was applied, as well as conventional synthesis, using cyclohexanone (or cyclopentanone), malononitrile (1:1 molar ratio) and 3–4 drops of glacial AcOH with DMF as a solvent and compound **1**, to give the pyrano[2,3-*f*]chromene derivatives **8a**,**b**, as indicated by elemental analysis and spectral data (Scheme 2, Table 1). Formation of the spiro compounds **8a**,**b** was proceeded according to the proposed mechanistic pathway (Chart 1).

**Table 1:** Physical data of the synthesized compounds **2**–**16**.

Compounds	Mol. Formula	Mol. Wt.	Time (min/h)		Yield (%)		Melting Point (°C)
			Microwave(min)	Conventional(h)	Microwave	Conventional	
**2**	C_10_H_6_O_4_	190.15	8	5	97	85	217–219
**3**	C_10_H_5_ClO_3_	208.60	-	1	96	75	145–147
**4**	C_10_H_7_ClO_2_	194.61	-	1	-	80	236–238
**5**	C_10_H_10_N_2_O_2_	190.20	9	4	97	85	126–128
**8a**	C_18_H_16_N_2_O_3_	308.12	9	4	98	78	167–169
**8b**	C_19_H_18_N_2_O_3_	322.36	10	5	95	82	151–153
**11**	C_19_H_13_N_3_O_5_S_2_	427.45	9	7	96	89	251–253
**13**	C_19_H_16_N_2_O_3_S	352.41	8	4	98	80	278–280
**16**	C_20_H_12_N_2_O_5_S_2_	452.46	10	5	97	70	211–213
**17**	C_20_H_15_N_3_O_3_S	377.42	8	6	96	80	182–184
**19**	C_27_H_19_N_5_O_4_S_3_	573.67	10	6	96	73	198–200
**20**	C_27_H_22_N_4_O_2_S_2_	498.62	10	7	95	85	217–219

**Method A**: Microwave-assisted synthesis: AcOH, DMF as a solvent, 120 °C, 8–10 min. **Method B**: Conventional synthesis: AcOH, DMF as a solvent, reflux 4–6 h.

The IR spectrum of product **8b**, as an example, revealed absorption bands at 1695, 2217 and 3361 cm*^−^*^1^ characteristic for C=O, C≡N and NH_2_ groups, respectively. Its ^1^H-NMR spectrum showed multiplet signals for protons of the methylene groups centered around δ 1.00–1.70 ppm in addition to the presence of two singlet signals, one at 2.43 ppm attributable to methyl protons and the other at 6.82 ppm, exchangeable with D_2_O, attributed to the NH_2_ protons. The mass spectrum of compound **8b** revealed a molecular ion peak at *m/z* 322 (M^+^, 57%), and a base peak was observed in the spectrum at *m/z* 176 (100%), which is compatible with its molecular formula C_19_H_18_N_2_O_3_.

4-Formylcoumarin **2** reacted with 5-amino-2-methylthieno[3,4-*d*]pyrimidin-4(3*H*)-one (**9**) and thioglycolic acid according to Method A and Method B, to give 5-(2-(6-hydroxy-2-oxo-2*H*-chromen-4-yl)-4-oxothiazolidin-3-yl)-2-methylthieno[3,4-*d*]pyrimidin-4(3*H*)-one (**11**) (Scheme 3, Table 1). Its IR spectrum displayed absorption bands at 1681–1708, 3156 and 3480 cm^−1^ due to three carbonyls, imino and hydroxyl groups, respectively. The mass spectrum of the product revealed a molecular ion peak at *m/z* 427 corresponding to its molecular formula C_19_H_13_N_3_O_5_S_2_. Its ^1^H-NMR spectrum shows five singlet signals at δ 2.43, 3.97, 6.46, 6.51, 6.61, 12.21 and 12.50 ppm due to the methyl, thiazolidinone-H5, thiophene-H5, coumarin-H3, thiazolidinone-H2, NH and OH groups, respectively, and multiplet signals in the δ 7.03–7.51 ppm region due to the aromatic protons.

**Method A**: Microwave-assisted synthesis: AcOH, DMF as a solvent, 120 °C, 8–10 min. **Method B**: Conventional synthesis: AcOH, DMF as a solvent, reflux 4–7 h.

Similarly, 3-((4-methyl-2-oxo-2*H*-chromen-6-yl)amino)-2-phenylthiazolidin-4-one (**13**) was synthesized via reaction of 6-hydrazinyl-4-methyl-2*H*-chromen-2-one (**5**) with benzaldehyde (**12**) and thioglycolic acid (**10**) under the previous conditions (Scheme 4, Table 1). In the corresponding IR spectrum an absorption band due to the (C=O*_str_*) of the thiazolidinone was observed at 1708 cm^−1^, and another (N-H*_str_*) band was found at 3280 cm^−1^. The ^1^H-NMR spectrum of **13** showed singlet signals of the cyclized thiazolidinone at 3.95 and 5.91 ppm corresponding to -CH_2_- in the ring and the NH proton, respectively. In its ^13^C-NMR spectrum the up field resonances of the carbonyl carbon were observed at 170.4 beside that of the other coumarin carbonyl at 160.1 ppm.

The reaction of thiazolidinones **11** or **13** with dimethylformamide-dimethylacetal (DMF-DMA) (**14**) and hydroxylamine (**15**) as potential precursors for thiazolo[5,4-*d*]isoxazoles was also investigated. Thus, a one-pot, microwave assisted as well as conventional synthesis, by using 5-(2-(6-hydroxy-2-oxo-2*H*-chromen-4-yl)-4-oxothiazolidin-3-yl)-2-methylthieno[3,4-*d*]pyrimidin-4(3*H*)-one (**11**) or 3-(4-methyl-2-oxo-2*H*-chromen-6-ylamino)-2-phenylthiazolidin-4-one (**13**) with dimethylformamide-dimethylacetal **(14**) and hydroxylamine (**15**) in DMF as a solvent containing 3–4 drops of glacial AcOH yielded 5-(5-(6-hydroxy-2-oxo-2*H*-chromen-4-yl)thiazolo[5,4-*d*]isoxazol-6(5*H*)-yl)-2-methyl-thieno[3,4-*d*]pyrimidin-4(3*H*)-one (**16**) or 4-methyl-6-(5-phenylthiazolo[5,4-*d*]isoxazol-6-ylamino)-2*H*-chromen-2-one (**17**), respectively (Scheme 5 and Scheme 6, Table 1).

**Method A**: Microwave-assisted synthesis: Glacial AcOH, DMF as a solvent, 120 °C, 8–10 min. **Method B**: Conventional synthesis: Glacial AcOH, DMF as a solvent, reflux 4–7 h.

**Method A**: Microwave-assisted synthesis: AcOH, DMF as a solvent, 120 °C, 8–10 min. **Method B**: Conventional synthesis: AcOH, DMF as a solvent, reflux 4–7 h.

The IR spectrum of **16** showed absorption bands at 3453, 3263, 1,696 and 1681 cm^−1^ corresponding to O-H*_str_*, N-H*_str_* and two C=O*_str_* functions, respectively. Its ^1^H-NMR spectrum showed two sharp singlet signals at δ 4.95 and 8.14 and two broad singlet signals at 10.56 and 12.52 characteristic of thiazole-H2, isoxazole-H3, NH and OH protons, respectively, besides a multiplet in the δ 7.28–7.80 ppm region distinctive for aromatic protons. Its mass spectrum showed a molecular ion peak at *m/z* 452, corresponding to its molecular formula (C_20_H_12_N_2_O_5_S_2_).

Moreover, the reactivity of the 4-thiazolidinone derivatives **11** and **13** a key intermediates for the synthesis of fused thiazolo[4,5-*d*]pyrimidine derivatives has been investigated. Thus, a one-pot three-component condensation reaction of 5-(2-(6-hydroxy-2-oxo-2*H*-chromen-4-yl)-4-oxothiazolidin-3-yl)-2-methylthieno[3,4-*d*]pyrimidin-4(3*H*)-one (**11**) or 3-(4-methyl-2-oxo-2*H*-chromen-6-ylamino)-3-phenylisothiazolidin-4-one (**13**) with benzaldehyde (**12**) and thiourea (**18**) proceeded smoothly in DMF containing 3-4 drops of glacial AcOH acid via microwave assisted as well as conventional synthesis to give 5-(2-(6-hydroxy-2-oxo-2*H*-chromen-4-yl)-7-phenyl-5-thioxo-5,6,7,7a-tetrahydrothiazolo[4,5-*d*]pyrimidin-3(2*H*)-yl)-2-methylthieno[3,4-*d*]pyrimidin-4(3*H*)-one (**19**) or 6-(2,7-diphenyl-5-thioxo-5,6,7,7a-tetrahydrothiazolo[4,5-*d*]pyrimidin-3(2*H*)-ylamino)-4-methyl-4a,8a-dihydro-2*H*-chromen-2-one (**20**), respectively, (Scheme 7 and Scheme 8, Table 1).

**Method A**: Microwave-assisted synthesis: AcOH, DMF as a solvent, 120 °C, 8–10 min. **Method B**: Conventional synthesis: AcOH, DMF as a solvent, reflux 4–7 h.

In the IR spectrum of the latter product absorption bands were observed at 1378, 1684 and 3250–3486 cm^−1^ corresponding to (C=S*_str_*), (C=O*_str_*) and (N-H*_str_*) vibrations, respectively. The ^1^H-NMR spectrum of **19** indicated the presence of five singlet signals at δ 2.41, 6.46, 6.51, 6.71, 12.20 and 12.52 ppm due the methyl, thiophene-H5, coumarin-H3, thiazole-H2, -NHCO- and OH groups, respectively, and two doublets centered around 3.41 and 4.21 ppm attributed to the –NH-C=S of the pyrimidine ring and pyrimidine-H6 respectively, and multiplet signals at 7.03–7.51 ppm due to aromatic protons. Its mass spectrum showed a molecular ion peak at *m/z* 573, corresponding to a molecular formula C_27_H_19_N_4_O_4_S_3_. The mass spectrum of **20** showed a molecular ion peak at *m/z* 498, corresponding to a molecular formula C_27_H_22_N_4_O_2_S_2_.

## 3. Experimental

### 3.1. General Information

6-Hydroxy-4-methyl-2*H*-chromen-2-one, dimethyformamide dimethylacetal (DMF-DMA), phosphoryl chloride, cyclohexanone, cyclopentanone, benzaldehyde, thioglycolic acid, glacial AcOH, thiourea and *N*,*N*-dimethylformamide (DMF) were purchased from Sigma Aldrich (Seelze, Germany). Reaction progress was monitored by TLC on silica gel precoated F254 Merck plates (Merck, Dublin, Ireland). Developed plates were examined with ultraviolet lamps (254 nm). All melting points were determined on a Gallenkamp Electrothermal melting point apparatus and are uncorrected, IR spectra were recorded as potassium bromide pellets using a FTIR Vector 22 spectrophotometer (Bruker, Manasquan, NJ, USA). ^1^H-NMR and ^13^C-NMR spectra were recorded in DMSO-*d*_6_ solvent, respectively, on a WP spectrometer (300 MHz for ^1^H-NMR and 75 MHz for ^13^C-NMR) (Bruker, Marietta, GA, USA), and the chemical shifts are reported in δ units downfield from TMS used as an internal standard. Mass spectra were recorded on a MS-5988 spectrometer at 70 e.v. (Hewlett Packard, Palo Alto, CA, USA). Elemental analysis was carried out at the Microanalytical Center of Cairo University, Egypt.

### 3.2. Synthesis

#### 3.2.1. General Procedure for Microwave-Assisted Synthesis of 6-Hydroxy-2-oxo-2*H*-chromene-4-carbaldehyde (**2**), Method A

An equimolar amount of 6-hydroxy-2-oxo-2*H*-chromene-4-carbaldehyde (**1**) (1 mmol) and selenium dioxide (1 mmol) was added in DMF (volume) along with 2-3 drops of glacial AcOH. This mixture was placed in a 100 mL round bottom flask and subjected to MW irradiation (800 W), at 120 °C temperature for 6 min. The completion of the reaction progress was monitored by using TLC (5% ethyl acetate-*n*-hexane). The product obtained was poured into crushed ice, filtered and washed with petroleum ether and ethyl acetate (1:4, 3 × 10 mL). The combined solvent extracts were concentrated *in vacuo*. The product was finally recrystallized from EtOH to afford product **2** in 97% yield.

#### 3.2.2. General Procedure for Conventional Synthesis of 6-Hydroxy-2-oxo-2*H*-chromene-4-carbaldehyde (**2**), Method B

6-Hydroxy-2-oxo-2*H*-chromene-4-carbaldehyde (**1**, 1 mmol) was dissolved in boiling DMF containing 2-3 drops of glacial AcOH, and to this boiling solution was added portionwise, with stirring, powdered selenium dioxide (1 mmol). After complete addition, boiling and stirring were continued for 3 h The completion of the reaction progress was monitored by using TLC (5% ethyl acetate-*n*-hexane). The product obtained was poured into crushed ice, filtered and washed with petroleum ether and ethyl acetate (1:4) (3 × 10 mL). The combined solvent extracts were concentrated *in vacuo*. The product was recrystallized from EtOH to obtain pure product **2** as a yellow solid, in 85% yield; mp 217–219 °C; IR (cm^−1^): 1695, 1710 (2C=O*_str_*), 3431 (O-H*_str_*); ^1^H-NMR: 6.70 (*s*, 1H, coumarin-H3, 7.70–7.98 (*m*, 3H, Ar-H), 10.45 (*s*, 1H, CHO) and 12.01 (*s*, 1H, OH). ^13^C-NMR: 91.1 (coumarin-C3), 119.8, 125.7, 126.6, 128.8, 133.7 and 150.1 (benzene), 162.4 (C=O) and 192.5 (CHO). MS (*m/z*%) = 190 ([M]^+^, 51%). Anal. Calcd. for C_10_H_6_O_4_ (190.15): C, 63.16; H, 3.18; N33.66%. Found: C, 63.01; H, 3.10; N, 33.46%.

#### 3.2.3. Synthesis of 6-Chloro-2-oxo-2*H*-chromene-4-carbaldehyde (**3**)

#### Microwave-Assisted Synthesis, Method A

Compound **3** was prepared according to the general procedure 3.2.1 (Method A) described for compound **2** via the reaction of an equimolar amount of 6-chloro-4-methyl-2*H*-chromen-2-one (**4**, 1 mmol) and selenium dioxide (1 mmol) to obtain pure product **3** in 96% yield.

#### Conventional Synthesis, Method B

Prepared according to the general procedure 3.2.2 (Method B) described for compound **2** via the reaction of an equimolar amount of 6-chloro-4-methyl-2*H*-chromen-2-one (**4**, 1 mmol) and selenium dioxide (1 mmol) to obtain pure product **3** in 75% yield.

#### Conventional Synthesis, Method C

To a stirred mixture of 6-hydroxy-2-oxo-2*H*-chromene-4-carbaldehyde (**2**, 1.90 g, 1 mmol) and anhydrous EtOH (30 mL) was added dropwise POCl_3_ (5 mL) at 5–10 °C. The reaction mixture was then stirred for an additional 1 h at room temperature and then heated for 2 h at 60 °C. After the reaction was completed, the mixture was poured onto crushed ice (200 g) under vigorous stirring. The mixture was kept overnight at 0 °C; resulted solid was collected by filtration and washed successively with water and then was air-dried to provide **3**, and finally recrystallized from EtOH, as an orange solid, in 60% yield; mp 145–147 °C; IR (cm^−1^): 1695–1710 (2C=O*_str_*); ^1^H-NMR: 6.70 (*s*, 1H, coumarin-H3, 7.70–7.98 (*m*, 3H, Ar-H) and 10.35 (*s*, 1H, CHO). ^13^C-NMR: 91.1 (coumarin-C3), 119.8, 125.7, 126.6, 128.8, 133.7 and 150.1 (benzene), 162.4 (C=O) and 189.5 (CHO). MS (*m/z* %) = 208 (M^+^, 71%). Anal. Calcd for C_10_H_5_ClO_3_ (208.60): C, 57.58; H, 2.42; Cl, 17.00; O, 23.01%. Found: C, 56.48; H, 2.22; Cl, 16.89; O, 22.81%.

#### 3.2.4. Synthesis of 6-Chloro-4-methyl-2*H*-chromen-2-one (**4**), Method C

This compound was prepared according to the general procedure 3.2.2.3 (Method C) described for compound **3** via the reaction of hydroxylcoumarin (**1**, 1 mmol) with POCl_3_ (5 mL) to afford **4** as a yellow solid which recrystallized from a mixture of EtOH/ DMF (3:1) as yellow crystals; in 80% yield; mp 236–238 °C; IR (cm^−1^): 1695-1710 (2 C=O*_str_*); ^1^H-NMR: 2.48 (*s*, 3H, CH_3_), 6.63 (*s*, 1H, coumarin-H3, 7.02–7.53 (*m*, 3H, Ar-H). ^13^C-NMR: 18.9 (Me), 115.6 (coumarin-C3), 119.8, 125.7, 126.6, 128.8, 133.7 and 150.1 (benzene), 161.4 (C=O). MS (*m/z* %) = 194 (M^+^, 45%). Anal. Calcd for C_10_H_7_ClO_2_ (194. 61): C, 61.72; H, 3.63; Cl, 18.22; O, 16.44%. Found: C, 61.67; H, 3.45; Cl, 18.13; O, 16.24%.

#### 3.2.5. Synthesis of 6-Hydrazinyl-4-methyl-2*H*-chromen-2-one (**5**)

#### Microwave-Assisted Synthesis, Method A

Prepared according to the general procedure 3.2.1 (Method A) described for compound **2** via the reaction of an equimolar amount of 6-chloro-4-methyl-2*H*-chromen-2-one (**4**, 1 mmol) and hydrazine hydrate (1 mmol) to obtain pure product **5** in 97% yield.

#### Conventional Synthesis of 6-Hydrazinyl-4-methyl-2*H*-chromen-2-one (**5**), Method B

A mixture of chlorocoumarin **2** (1.90 g, 1 mmol) and hydrazine hydrate (0.5 mL, 1 mmol) in EtOH (30 mL) containing triethylamine (0.1 mL) was refluxed at 80 °C for 4 h, the reaction mixture was concentrated under reduced pressure and the residue washed with acidified cold water and then triturated with MeOH. The pale yellow product was filtered, washed well with MeOH, in 85% yield; mp 126–128 °C. IR: 1695 (C=O***_str_***), 3212–3423 (NH***_str_*** and NH_2_***_str_***). ^1^H-NMR (DMSO-*d_6_*): 1.32 (*s*, 3H, CH_3_), 4.28 (br., *s*, 2H, NH_2_, D_2_O-exchangeable), 7.21–7.65 (*m*, 3H, Ar-H), 9.21 (br., *s*, 1H, NH, D_2_O-exchangeable); ^13^C-NMR (DMSO-*d_6_*): 19.7 (Me), 112.6 (coumarin-C3), 119.8, 125.7, 126.6, 128.8, 133.7 and 150.1 (benzene), 161.8 (C=O). Ms: *m/z* 190 (M^+^, 70%). Anal. Calcd for C_10_H_10_N_2_O_2_ (190.20): C 63.15, H 5.30, N 14.73%. Found: C 63.01, H 5.12, N 14.54%.

#### 3.2.6. General Procedure for Microwave-Assisted Synthesis of Spiro Compounds **8a** and **8b**, Method A

An equimolar amount of hydroxycoumarin (**1**, 1 mmol), cyclopentanone (**6a**, 1 mmol) or cyclohexanone (**6b**, 1 mmol) and malononitrile (**7**, 1 mmol) were reacted according to the general procedure 3.2.1 (Method A) to give **8a**,**b**. in 98% and 95% yields respectively.

#### 3.2.7. General Procedure for Conventional Synthesis of Spiro Compounds **8a** and **8b**, Method B

An equimolar amount of hydroxylcoumarin (**1**, 1 mmol), cyclopentanone(**6a**, 1 mmol) or cyclohexanone (**6b**, 1 mmol) and malononitrile (**7**, 1 mmol) were reacted according to the general procedure 3.2.2 (Method B) to give **8a**,**b**. in 78% and 82% yields respectively.

*2'-Amino-10'-methyl-8'-oxo-5'H-spiro[cyclopentane-1,1'-pyrano[3,2-f]chromene]-3'-carbonitrile* (**8a**). Brown crystals (EtOH/DMF (1:1)); mp 167–169 °C; IR (cm^−1^): 1695 (C=O***_str_***), 2210 (CN***_str_***), 3363 (NH_2***str***_); ^1^H-NMR: 1.00–1.07 (*m*, 3H, Cy-H), 1.24–1,35 (*m*, 2H, Cy-H), 1.53–1.70 (*m*, 5H, Cy-H), 2.43 (*s*, 3H, CH_3_), 6.23 (*s*, 1H, coumarin-H3), 6.82 (br., *s*, 2H, NH_2_, D_2_O-exchangeable), 7.42 (*d*, 1H, *J* = 4, Ar-H), 7.53 (*d*, 1H, *J* = 4, Ar-H). ^13^C-NMR: 23.7 (Me), 20.5, 24.4, 26.4, 35.2 (Cy-C), 112.6 (coumarin-C3), 117.4 (CN), 119.8, 125.7, 126.6, 128.8, 133.7, 150.1 (benzene), 67.1, 175.6 (Py-C), 160.8 (C=O). MS (*m/z*%) = 308 (M^+^, 25%). Anal. Calcd for C_18_H_16_N_2_O_3_ (308.12): C, 70.12; H, 5.23; N, 9.09%. Found: C, 70.02; H, 5.11; N, 8.99%.

*3'-Amino-10'-methyl-8'-oxo-8'H-spiro[cyclohexane-1,1'-pyrano[3,2-f]chromene]-2'-carbonitrile* (**8b**). Reddish brown crystals (EtOH/DMF (1:1)); mp 151–153 °C; IR (cm^−1^): 1695 (C=O***_str_***), 2217 (CN***_str_***), 3361 (NH_2***str***_); ^1^H-NMR: 1.00–1.05 (*m*, 3H, Cy-H), 1.53–1.65 (*m*, 5H, Cy-H), 1.66–1.70 (*m*, 2H, Cy-H), 2.43 (*s*, 3H, CH_3_), 6.43 (*s*, 1H, coumarin-H3, 6.72 (br., *s*, 2H, NH_2_, D_2_O-exchangeable), 7.42 (*d*, 1H, *J* = 4, Ar-H), 7.53 (*d*, 1H, *J* = 4, Ar-H). ^13^C-NMR: 23.7 (Me), 20.5, 24.4, 26.4, 35.2 (Cy-C), 112.6 (coumarin-C3), 117.4 (CN),119.8, 125.7, 126.6, 128.8, 133.7, 150.1 (benzene), 67.1, 175.6 (Py-C), 160.8 (C=O). MS (*m/z*%) = 322 (M^+^, 57%). Anal. Calcd for C_19_H_18_N_2_O_3_ (322.36): C, 70.69; H, 5.63; N, 8.69%. Found: C, 70.72; H, 5.46; N, 8.76%.

#### 3.2.8. Microwave-Assisted Synthesis of 5-(2-(6-Hydroxy-2-oxo-2*H*-chromen-4-yl)-4-oxothiazolidin-3-yl)-2-methylthieno[3,4-*d*]pyrimidin-4(3*H*)-one (**11**)

An equimolar amount of 6-hydroxy-2-oxo-2*H*-chromene-4-carbaldehyde (**2**, 1 mmol), 5-amino-2-methylthieno[3,4-*d*]pyrimidin-4(3*H*)-one (**9**, 1 mmol) and thioglycolic acid (**10**, 1 mmol) was reacted according to the general procedure 3.2.1 (Method A) to give **11**.

#### 3.2.9. Conventional Synthesis of Compound **11**

An equimolar amount of 6-hydroxy-2-oxo-2*H*-chromene-4-carbaldehyde (**2**, 1 mmol), 5-amino-2-methylthieno[3,4-*d*]pyrimidin-4(3*H*)-one (**9**, 1 mmol) and thioglycolic acid (**10**, 1 mmol) were reacted according to the general procedure 3.2.2 (Method B) to give **11** as a yellow solid, mp 251–253 °C; IR (cm^−1^): 1681–1708 (3 C=O***_str_***), 3156 (*br*, N-H***_str_***), 3480 (O-H***_str_***); ^1^H-NMR): 2.43 (*s*, 3H, CH_3_), 3.97 (*s*, 2H, CH_2_ of thiazolidine), 6.46 (*s*, 1H, thiophene methine), 6.51 (*s*, 1H, Coum-H3), 6.61 (*s*, 1H, thiazolidine-H2), 7.03–7.51 (*m*, 3H, Ar-H), 12.21 (*s*, br., 1H, -NH), 12.50 (*s*, 1H, OH). ^13^C-NMR: 21.8 (CH_3_), 33.8 (C-5-thiazolidine), 117.4 (C-2-thiophene), 124 (C-3-thiophene), 126 (C-4-thiophene), 152.8 (C-5-thiophene), 161.2 (C-2-thiazolidine), 154.8 (C-2-pyrimidine), 161.0, 162.4, 171,2 (3C=O) and 119.8, 125.7, 126.6, 128.6, 133.7 and 150.1 (Ph). MS (*m/z* %) = 427 (M^+^, 60%). Anal. Calcd for C_19_H_13_N_3_O_5_S_2_ (427.45): C, 53.39; H, 3.07; N, 9.83%. Found: C, 53.21; H, 3.01; N, 9.67%.

#### 3.2.10. Microwave-Assisted Synthesis of 3-((4-Methyl-2-oxo-2*H*-chromen-6-yl)amino)-2-phenyl-thiazolidin-4-one (**13**)

An equimolar amount of 6-hydrazinyl-4-methyl-2*H*-chromen-2-one (**5**, 1 mmol), benzaldehyde (**12**, 1 mmol), and thioglycolic acid (**10**, 1 mmol) were reacted to according to the general procedure 3.2.1 (Method A) to give **13**.

#### 3.2.11. Conventional Synthesis of Compound **13**

Equimolar amounts of 6-hydrazinyl-4-methyl-2*H*-chromen-2-one (**5**, 1 mmol), benzaldehyde (**12**, 1 mmol), and thioglycolic acid (**10**, 1 mmol) were reacted according to the general procedure 3.2.2 (Method B) to give **13** as a brown solid, mp 278–280 °C; IR (cm^−1^): 1681-1708 (2 C=O***_str_***), 3280 (*br*, N-H*_str_*); ^1^H-NMR: 2.43 (*s*, 3H, CH_3_), 3.95 (*s*, 2H, CH_2_ of thiazolidine), 6.23 (*s*, 1H, Coum-H3), 5.91 (*s*, br., 1H, -NH-), 5.91 (*s*, 1H, thiazolidine-H2), 7.03–7.51 (*m*, 8H, Ar-H). ^13^C-NMR: 19.1 (CH_3_), 36.0 (C-5-thiazolidine), 58.2 (C-2-thiazolidine), 160.1, 170.4 (2C=O) and 119.8, 125.7, 126.6, 128.6 and 133.7, 150.1 (Ph). MS (*m/z* %) = 352 (M^+^, 65%). Anal. Calcd for C_19_H_16_N_2_O_3_S (352.41): C, 64.76; H, 4.58; N, 7.95%. Found: C, 64.56; H, 4.32; N, 7.87%.

#### 3.2.12. General Procedure for Microwave-Assisted Synthesis of Thiazolo[5,4-*d*]isoxazole Derivatives **16** and **17**, Method A

Equimolar amounts of 5-(2-(6-hydroxy-2-oxo-2*H*-chromen-4-yl)-4-oxothiazolidin-3-yl)-2-methylthieno[3,4-*d*]pyrimidin-4(3*H*)-one (**11**, 1 mmol) or 3-(4-methyl-2-oxo-2*H*-chromen-6-ylamino)-2-phenylthiazolidin-4-one (**13**, 1 mmol), dimethylformamide-dimethylacetal (DMF-DMA) (**14**, 1 mmol), and hydroxylamine (**15**, 1 mmol) were reacted according to the general procedure 3.2.1 (Method A) to afford pure products **16** and **17**, respectively.

#### 3.2.13. General Procedure for Conventional Synthesis of Synthesis of Thiazolo[5,4-*d*]isoxazole Derivatives **16** and **17**, Method B

Equimolar amounts of 5-(2-(6-hydroxy-2-oxo-2*H*-chromen-4-yl)-4-oxothiazolidin-3-yl)-2-methylthieno[3,4-*d*]pyrimidin-4(3*H*)-one (**11**, 1 mmol) or 3-(4-methyl-2-oxo-2*H*-chromen-5-ylamino)-2-phenylthiazolidin-4-one (**13**), dimethylformamide-dimethylacetal (DMF-DMA) (**14**, 1 mmol), and hydroxylamine (**15**, 1 mmol) were reacted according to the general procedure 3.2.2 (Method B) to obtain pure products **16** and **17**, respectively.

*5-(5-(6-Hydroxy-2-oxo-2H-chromen-4-yl)thiazolo[5,4-d]isoxazol-6(5H)-yl)-2-methylthieno-[3,4-d]-pyrimidin-4(3H)-one* (**16**). Pale brown solid, mp 210–213 °C; IR (cm^−1^): 1681, 1696 (2 C=O***_str_***), 3263 (br, N-H***_str_***), 3453 (O-H***_str_***); ^1^H-NMR: 2.71 (*s*, 3H, CH_3_), 4.95 (*s*, 1H, thiazole-H2), 6.42 (*s*, 1H, methine of thiophene-H5), 7.28–7.80 (*m*, 3H, Ar-H), 8.14 (*s*, 1H, isoxazole-H3), 10.56 (*s*, br., 1H, -NH), 12.52 (*s*, 1H, OH). ^13^C-NMR: 21.4 (CH_3_), 70.71 (C-2-thiazole), 100.0 (C-5-thiazole), 125.0 (C-4-thiophene), 129.4 (C-5-thiophene), 137.0 (C-2-thiophene), 142.0 (C-3-thiophene), 150.0 (C-3-isoxazole), 154.5 (C-2-pyrimidine), 160.8, 161.0 (2C=O) and 109.8, 125.7, 126.6, 128.6 and 133.7, 150.1 (Ph). MS (*m/z* %) = 452 (M^+^, 25%). Anal. Calcd for C_20_H_12_N_4_O_5_S_2_ (452.46): C, 53.09; H, 2.67; N, 12.38%. Found: C, 53.01; H, 2.56; N, 12.23%.

*4-Methyl-6-(5-phenylthiazolo[5,4-d]isoxazol-6-ylamino)-4a,8a-dihydro-2H-chromen-2-one* (**17**). Brown solid, mp 182–184 °C; IR (cm^−1^): 1681 (C=O***_str_***), 3263 (*br*, N-H***_str_***); ^1^H-NMR: 2.43 (*s*, 3H, CH_3_), 4.95 (*s*, 1H, thiazole-H2), 6.43 (*s*, 1H, Coum-H3), 5.93 (*s*, br., 1H, -NH-), 7.03–7.51 (*m*, 8H, Ar-H), 8.17 (*s*, 1H, isoxazole-H3). ^13^C-NMR: 19.1 (CH_3_), 72.4 (C-2-thiazole), 100.0 (C-5-thiazole), 150.0 (C-3-isoxazole), 160.8 (C=O) and 119.8, 125.7, 126.6, 128.6 and 133.7, 150.1 (Ph). MS (*m/z* %) = 377 (M^+^, 35%). Anal. Calcd for C_20_H_15_N_3_O_3_S (377.42): C, 63.65; H, 4.01; N, 11.13%. Found: C, 63.53; H, 3.98; N, 11.02%.

#### 3.2.14. General Procedure for Microwave-Assisted Synthesis of Thiazolo[4,5-*d*]pyrimidine Derivatives **19** and **20**, Method A

An equimolar amount of 5-(2-(6-hydroxy-2-oxo-2*H*-chromen-4-yl)-4-oxothiazolidin-3-yl)-2-methylthieno[3,4-*d*]pyrimidin-4(3*H*)-one (**11**, 1 mmol) or 3-(4-methyl-2-oxo-2*H*-chromen-6-ylamino)-2-phenylthiazolidin-4-one (**13**), benzaldehyde **12** (1 mmol) and thiourea **18** was reacted according to the general procedure 3.2.1 (Method A) to give pure products **19** and **20**, respectively.

#### 3.2.15. General Procedure for Conventional Synthesis of Thiazolo[5,4-*d*]isoxazole Derivatives **19** and **20**, Method B

Equimolar amount of 5-(2-(6-hydroxy-2-oxo-2*H*-chromen-4-yl)-4-oxothiazolidin-3-yl)-2-methylthieno[3,4-*d*]pyrimidin-4(3*H*)-one (**11**, 1 mmol) or 3-(4-methyl-2-oxo-2*H*-chromen-6-ylamino)-2-phenylthiazolidin-4-one (**13**), benzaldehyde (**12**) and thiourea (**18**) was reacted according to the general procedure 3.2.1 (Method A) to afford pure products **19** and **20**, respectively.

5-(2-(6-Hydroxy-2-oxo-2*H*-chromen-4-yl)-7-phenyl-5-thioxo-5,6,7,7a-tetrahydrothiazolo[4,5-*d*]pyrimidin-3(2*H*)-yl)-2-methylthieno[3,4-*d*]pyrimidin-4(3*H*)-one (**19**). Yellow solid, mp 198–200 °C; IR (cm^−1^): 1378 (C=S*_str_*), 1684 (C=O*_str_*), 3250–3486 (*br*, 2NH*_str_*); ^1^H-NMR: 2.41 (*s*, 3H, CH_3_), 3.20 (*d*, 1H, thiazole-H5), 3.41 (*d*, 1H, NH-CS-), 4.21 (*d*, 1H, pyrimidine-H6), 6.46 (*s*, 1H, thiophene-H5), 6.51 (*s*, 1H, Coum-H3), 6.71 (*s*, 2H, thiazole-H2), 7.03-7.51 (*m*, 8H, Ar-H), 12.20 (*s*, br., 1H, pyrimidine-NH), 12.52 (*s*, 1H, OH). ^13^C-NMR: 21.4 (CH_3_), 33.8 (C-5-thiazole), 117.4 (C-2-thiophene), 124 (C-3-thiophene), 126 (C-4-thiophene), 152.8 (C-5-thiophene), 161.2 (C-2-thiazole), 154.8 (C-2-pyrimidine), 160.8, 161.0 (2C=O) and 119.8, 125.7, 126.6, 128.6, 133.7, 150.1 (Ph), 187.0 (C=S). MS (*m/z*%) = 573 (M^+^, 45%). Anal. Calcd for C_27_H_19_N_5_O_4_S_3_ (573.67): C, 56.53; H, 3.34; N, 12.21; S, 16.77%. Found: C, 56.27; H, 3.21; N, 11.89; S, 16.46%.

*6-(2,7-Diphenyl-5-thioxo-5,6,7,7a-tetrahydrothiazolo[4,5-d]pyrimidin-3(2H)-ylamino)-4-methyl-2H-chromen-2-one* (**20**). Yellow crystals, mp 217–219 °C; IR (cm^−1^): 1378 (C=S*_str_*), 1684 (C=O*_str_*), 3250–3486 (*br*, 2NH*_str_*); ^1^H-NMR: 2.43 (*s*, 3H, CH_3_), 3.20 (*d*, 1H, thiazole-H5), 3.41 (*d*, 1H, pyrimidine-NH), 4.2 (*d*, 1H, pyrimidine-H6), 4.95 (*s*, 2H, thiazole-H2), 6.23 (*s*, 1H, Coum-H3), 5.91 (*s*, br., 1H, -NH-), 7.03–7.51 (*m*, 13H, Ar-H). ^13^C-NMR: 19.4 (CH_3_), 68.0 (C-2-thiazole), 164.0 (C-5-thiazole), 160.8 (C=O) and 119.8, 125.7, 126.6, 128.6 and 133.7, 150.1 (2Ph), 187.0 (C=S). MS (*m/z*%) = 498 (M^+^, 70%). Anal. Calcd for C_27_H_22_N_4_O_2_S_2_ (489.62): C, 65.04; H, 4.45; N, 11.24; S, 12.86%. Found: C, 64.86; H, 4.34; N, 11.12; S, 12.67%.

### 3.3. Antimicrobial Evaluation

Some strains of bacteria and fungi were obtained from Assiut University Mycological Center (AUMC) and other strains were obtained from Aswan Teaching Hospital, Aswan, Egypt. All the synthesized compounds were screened for their *in vitro* antimicrobial activity, against three Gram positive bacteria; (BS): *B. subtilis* (MTCC 443); (CT): *C. tetani* (MTCC 449); (SP): *S. pneumoniae* (MTCC 1936); three Gram negative bacteria; (EC): *E. coli* (MTCC 440); (ST): *S. typhi* (MTCC 98); (VC): *V. cholerae* (MTCC 3906); and two fungal strains; (AF): *A. fumigates* (MTCC 3008); (CA): *C. albicans* (MTCC 227). The results are presented in Table 1, expressed in the form of *MIC* in μg mL. The antibacterial activity of compounds was monitored by observing their Minimum Inhibitory Concentration (MIC, μg/mL) as previously mentioned by broth dilution method [33] with A: ampicillin; B: ciprofloxacin; C: norfloxacin; D: chloramphenicol as control drugs. The antifungal study was carried out by the standard agar dilution method with E: nystatin and F: griseofulvin as control drugs, DMSO, which exhibited no activity against any of the used organisms, was used as a blank, (Table 2).

An examination of the data prescribed in Table 1 revealed that, some of the compounds were more potent or equipotent to the standard drugs against the Gram-positive bacteria *C. tetani* and a few against *S. pneumoniae* and *B. subtilis*. Against the Gram-positive bacteria *B. subtilis*, compound **17** (*MIC* = 65.5 μg·mL^−1^) was found to be more potent, whereas **2**, **4**, **8b**, **13**, and **20** (*MIC* = 250 μg·mL^−1^) shows comparable activity to ampicillin (*MIC* = 250 μg·mL^−1^). Moreover, compound **17** (*MIC* = 65.5 μg·mL^−1^) was found to more active as compared to norfloxacin (*MIC* = 100 μg·mL^−1^). Against *C. tetani*, compounds **3**, **11**, **17** and **19** (*MIC* = 100 μg/mL), and **4**, **5**, **8a** and **13** (*MIC* = 200 μg·mL^−1^) were found to be more potent, whereas **20** (*MIC* = 250 μg·mL^−1^) showed comparable activity to ampicillin (*MIC* = 250 μg·mL^−1^), while compounds **3**, **11**, **17** and **19** (*MIC* = 100 μg·mL^−1^) were equally potent as compared to ciprofloxacin (*MIC* = 100 μg·mL^−1^). Against *S. pneumoniae*, compound **16** (*MIC* = 50 μg·mL^−1^) showed comparable activity to chlormphenicol and ciprofloxacin (*MIC* = 50 μg·mL^−1^).

**Table 2:** Antimicrobial activity of compounds **1**–**20** (minimum inhibitory concentration (*MIC*) μg mL).

Compound	Gram-Positive Bacteria			Gram-Negative Bacteria			Fungal Species	
	(BS)	(CT)	(SP)	(EC)	(ST)	(VC)	(AF)	(CA)
**1**	500	500	500	250	500	500	1000	>1000
**2**	250	500	250	500	500	100	500	100
**3**	1000	100	500	250	500	200	250	100
**4**	250	200	250	500	250	200	500	250
**5**	500	200	500	250	250	200	500	500
**8a**	500	200	500	100	500	250	250	250
**8b**	250	500	250	100	100	250	1000	500
**11**	500	100	500	250	65.5	250	1000	1000
**13**	250	200	250	250	250	200	500	250
**16**	500	500	50	250	500	500	1000	500
**17**	65.5	100	250	100	65.5	200	1000	1000
**19**	500	100	500	200	500	200	500	500
**20**	250	250	500	100	65.5	250	250	250
**A**	250	250	100	100	100	100	0	0
**B**	50	100	50	25	25	25	0	0
**C**	100	50	10	10	10	10	0	0
**D**	50	50	50	50	50	50	0	0
**E**	0	0	0	0	0	0	100	100
**F**	0	0	0	0	0	0	100	500

A: ampicillin; B: ciprofloxacin; C: norfloxacin; D: chloramphenicol; E: nystatin; F: griseofulvin. “0” represents “not tested”.

Towards the Gram-negative strain *E. coli*, compounds **8a**, **8b**, **17** and **20** (*MIC* = 100 μg·mL^−1^) showed comparable activity to ampicillin (*MIC* = 100 μg·mL^−1^). Compounds **11**, **17** and **20** (*MIC* = 65.5 μg·mL^−1^) were more potent, whereas **8b** (*MIC* = 100 μg·mL^−1^) showed comparable activity to ampicillin (*MIC* = 100 μg·mL^−1^) towards *S. typhi.* Also the compound **20** (*MIC* = 100 μg/mL^−1^) show comparable activity, to ampicillin (*MIC* = 100 μg/mL^−1^) towards *V. cholerae*.

Against the fungal pathogen *C. albicans*, compounds **3** (*MIC* = 100 μg·mL^−1^) **4**, **8a**, **13** and **20** (*MIC* = 250 μg·mL^−1^) showed good to excellent activity, whereas **2**, **5**, **8b**, **16** and **19** (*MIC* = 500 μg·mL^−1^) were equipotent to griseofulvin (*MIC* = 500 μg·mL^−1^). Compound **3** (*MIC* = 100 μg·mL^−1^) was found equipotent to nystatin towards *C. albicans.* The remaining compounds showed moderate to good activity in the inhibition of the growth of bacterial pathogens and were all less effective than the standard drugs.

## 4. Conclusions

In summary, an efficient synthesis of some new 2*H*-chromene derivatives **1**–**20** bearing the phenylthiazolidinone nucleus via a facile one-pot three-component reaction under microwave irradiation as well as conventional chemical synthesis processes has been reported. Most of the synthesized compounds showed mild to moderately active against the *C. tetani*, a gram positive strain and *E. coli*, a gram negative strain. The antifungal activity of the compounds shows that most of the compounds were more potent against *C. albicans* than against *A. fumigatus*. Compounds **3**, **4**, **8a**, **13** and **20** exhibited remarkable antifungal activity against *C. albicans*.

## References

[1] Domling A. (2006). Recent Developments in Isocyanide Based Multicomponent Reactions in Applied Chemistry. Chem. Rev..

[2] Lafitte D., Lamour V., Tsvetkov P., Markov A.A., Deprez M., Klich P., Moras D., Briand C., Gilli R. (2002). DNA gyrase intgeraction with coumarin-based inhibitors-the role of the hydroxyl benzoate isopentenyl moiety and the 5'-methyl group of the noviose. Biochemistry.

[3] Hurry R.G., Cortz C., Ananthanaraxan T.P., Schmolka S. (1998). A new coumarin synthesis and its utilization for the synthesis of polycyclic coumarin compounds with anticarcinogenic properties. J. Org. Chem..

[4] Hafez E.A.A., Elnagdi M.H., Elagamey A.G.A., EL-Taweel F.M.A.A. (1987). Nitriles in Heterocyclic Synthesis: Novel Synthesis of Benzo[*c*]Coumarin and of Benzo[*c*]Pyrano[3,2-*c*]Quinoline Derivatives. Heterocycles.

[5] Tanabe A., Nakashima H., Yoshida O., Yamamoto N., Tenmyo O., Oki T. (1988). Inhibitory Effect of New Antibiotic, Pradimicin A on Infectivity, Cytopathic Effect and Replication of Human Immunodeficiency Virus *in Vitro*. J. Antibiot..

[6] Shijay G., Cheng H.T., Chi T., Ching-Fa Y. (2008). Fluoride Ion Catalyzed Multicomponet Reactions for Efficient Synthesis of 4*H*-Chromene and *N*-Arylquinoline Derivates in Aqueous Media. Tedrahedron.

[7] Balaji P.N., Lakshmi L.K., Mohan K., Revathi K., Chamundeswari A., Indrani P.M. (2012). *In-vitro* anti-inflammatory and antimicrobial activity of synthesized some novel pyrazole derivatives from coumarin chalcones. Der Pharmacia Sinica.

[8] Bayer T.A., Schafer S., Breyh H., Breyhan O., Wirths C., Treiber G.A. (2006). A Vicious Circle: Role of Oxidative Stress, Intraneuronal Aβ and Cu in Alzheimer’s Disease Multhaup. Clin. Neuropathol..

[9] Fokialakis N., Magiatis P., Chinou L., Mitaka S., Tillequin F., Megistoquinones I (2002). Two Quinoline Alkaloids with Antibacterial Activity from the Bark of Sarcomelicope megistophylla. Chem. Pharm. Bull..

[10] Beagley P., Blackie M.A.L., Chibale K., Clarkson C., Meijboom R., Moss J.R., Smith P., Su H. (2003). Synthesis and Antiplasmodial Activity *in Vitro* of New Ferrocene-Chloroquine Analogues. Dalton Trans..

[11] Morgan L.R., Jursic B.S., Hooper C.L., Neumann D.M., Thangaraj K., Leblance B. (2002). Anticancer Activity for 4,4-Dihydroxybenzophenone-2,4-Dinitrophenylhydrazone (A-007) Analogues and Their Abilities to interact with Lymphoendothelial Cell Surface Markers. Bioorg. Med. Chem. Lett..

[12] Bonsignore L., Loy G., Secci D., Calignano A. (1993). Synthesis and Pharmacological Activity of 2-oxo-(2*H*) 1-Benzopyran-3-Carboxamide Derivatives. Eur. J. Med. Chem..

[13] Cannon J.G., Khonji R.R. (1975). Centrally Acting Emetics. 9. Hofmann and Emde Degradation Products of Nuciferine. J. Med. Chem..

[14] Biot C., Glorian G., Maciejewski L.A., Brocard J.S., Domarle O., Blampain G., Blampain G., Blampain P., Georges A.J., Abessolo H. (1997). Synthesis and Antimalarial Activity *in Vitro* and *in Vivo* of a New Ferrocene-Chloroquine Analogue. J. Med. Chem..

[15] Abdel-Galil F.M., Riad B.Y., Sherif S.M., Elnagdi M.H. (1982). Activated Nitriles in Heterocyclic Synthesis: A Novel Synthesis of 4-Azoloyl-2- Aminoquinolines. Chem. Lett..

[16] Shaker R.M. (1996). Synthesis and Reactions of Some New 4*H*-Pyrano[3,2-*c*]benzopyran-5-One Derivatives and Their Potential Biological Activities. Pharmazie.

[17] Balalaie S., Abdolmohammadi S. (2007). Novel and Efficient Catalysts for the One-Pot Synthesis of 3,4-Dihydropyrano[*c*]Chromene Derivatives in Aqueous Media. Tetrahedron Lett..

[18] Kidwai M., Saxena S. (2006). Convenient Preparation of Pyrano Benzopyranes in Aqueous Media. Synth. Commun..

[19] Heravi M.M., Alimadadi J.B., Derikvand F., Bamoharram F.F., Oskooie H.A. (2008). Three Component, One-Pot Synthesis of Dihydropyrano[3,2-*c*]Chromene Derivatives in the Presence of H_6_P_2_W_18_O_62_·18H_2_O as a Green and Recyclable Catalyst. Catal. Commun..

[20] Seifi M., Sheibani H. (2008). High Surface Area MgO as a Highly Effective Heterogeneous Base Catalyst for Three-Component Synthesis of Tetra hydro benzopyran and 3,4-Dihydropyrano[*c*]chromene Derivatives in Aqueous Media. Catal. Lett..

[21] Khurana J.M., Kumar S. (2009). Tetra Buthyl Ammonium Bromide (TBAB): A Neutral and Efficient Catalyst for the Synthesis of Biscoumarin and 3,4-Dihydopyrano[*c*]Chromene Derivatives in Water and Solvent-Free Conditions. Tetrahedron Lett..

[22] Dobaria A.V., Patel J.R., Padaliya J.V., Parekh H.H. (2001). Thiazolidinones bearing chloroquinoline nucleus as potential antimicrobial agents. Indian J. Heterocycl. Chem..

[23] Deepak K., Bux F.B., Varsh P., Arun S. (2012). Thiazolidinone: Synthesis and biological studies. Der Pharma Chemica.

[24] Shweta T.D., Pratima R.P.S., Toraskar M.P. (2010). Synthesis and antimicrobial evaluation of some 4-substituted thiazolidinone derivatives. Der Pharma Chemica.

[25] Raghav M., Isha T., Sachin S., Jha K.K. (2012). Facile synthesis of thiazolidinones bearing thiophene nucleus as antimicrobial agents. Der Pharma Chemica.

[26] Aamer S., Naeem A., Ulrich F. (2007). Synthesis and Antibacterial Activity of some Novel 2-Aroylimino-3-aryl-thiazolidin-4-ones. J. Braz. Chem. Soc..

[27] El Azab I.H., El Rady E.A. (2012). Facile and Simple Synthesis of some New Polyfunctionally Heterocyclic Derivatives: Incorporating 2-Imino-2*H*-Chromene Moiety. J. Heterocycl. Chem..

[28] El Rady E.A., El Azab I.H. (2012). Reactivity of β-enamino ester of benzo[*f*]chromene: One pot synthesis of isolated and fused heterocyclic derivatives of benzo[*f*]chromene. Eur. J. Chem..

[29] El Azab I.H. (2013). Synthesis of Some New Benzo[*b*][1,4]diazepine Based Heterocycles. J. Heterocycl. Chem..

[30] Azab I.H., kenzy N.A.A. (2014). Synthesis of Fused- Isolated Azoles and *N*-Heteroaryl Derivatives Based on 2-Methyl-3,4-dihydrothieno[3,4-*d*]pyrimidin-5-amine. Synth. Commun..

[31] El Azab I.H., El Rady E.A. (2014). Simple Method for Synthesis of Isolated Heterocyclic Compounds: Incorporating 2-(2-Bromoacetyl)-isoindoline-1,3-dione and 2-(2-Cyanoacetyl)isoindo-line-1,3-dione. Indian J. Chem..

[32] Pal R., Handa R.N., Pujari H.K. (1992). Heterocyclic systems containing bridgehead nitrogen atom: Part LXIII. Reaction of 1-Methyl-7,8,10,11-tetraazaspiro[5,5]undecane-9-thione with bi functional compounds. Indian J. Chem. Soc..

[33] Cappuccino J.G., Sherman N. (1999). Microbiology- A Laboratory Manual.

