# *Correction:* El Azab, I.H., *et al*. Microwave-Assisted Synthesis of Novel 2*H*-Chromene Derivatives Bearing Phenylthiazolidinones and Their Biological Activity Assessment. *Molecules* 2014, *19*, 19648-19664

**DOI:** 10.3390/molecules20022835

**Published:** 2015-02-09

**Authors:** Islam H. El Azab, Mohamed M. Youssef, Amin M. Amin

**Affiliations:** 1 Chemistry Department, Faculty of Science, Taif University, Al-Haweiah, P.O. Box 888, Taif 21974, Saudi Arabia; 2 Chemistry Department, Faculty of Science, Aswan University, Aswan 81528, Egypt; 3 Chemistry Department, Faculty of Science, Cairo University, Giza 12613, Egypt; 4 Chemistry Department, Faculty of Science, Suez Canal University, Ismailia 41522, Egypt

The authors wish to revise the Author Affiliation section of the title paper, published in *Molecules* [1], (doi:10.3390/molecules191219648, website: http://www.mdpi.com/1420-3049/19/12/19648). To recognize the fact that the research described was performed in part at the facilities of Taif University and to acknowledge that institution’s generous financial support, the first two entries should be changed from:

^1^  Chemistry Department, Faculty of Science, Aswan University, Aswan 81528, Egypt
^2^  Chemistry Department, Faculty of Science, Taif University, Al-Haweiah, P.O. Box 888, Taif 21974, Saudi Arabia

to read as follows:

^1^  Chemistry Department, Faculty of Science, Taif University, Al-Haweiah, P.O. Box 888, Taif 21974, Saudi Arabia
^2^  Chemistry Department, Faculty of Science, Aswan University, Aswan 81528, Egypt
